# Identification and Characterization of a Splicing Variant in the 5′ UTR of the Human* TLR5* Gene

**DOI:** 10.1155/2017/8727434

**Published:** 2017-08-29

**Authors:** Thi Xoan Hoang, Cao Nguyen Duong, Jae Young Kim

**Affiliations:** Department of Life Science, Gachon University, Seongnam, Gyeonggi-do 461-701, Republic of Korea

## Abstract

Toll-like receptors (TLRs) are essential components of the innate immune system. TLR5 is the receptor for flagellin, the principal protein component of bacterial flagella. The* TLR5* gene has 6 exons. In an RT-PCR analysis, we found long* TLR5 *transcripts, in addition to those of the expected size (short* TLR5* transcripts). A sequence analysis revealed that the long* TLR5* transcripts contain a new exon of 94 nucleotides located between previously reported exons IV and V in the 5′ untranslated region (5′ UTR). A real-time PCR analysis of the two alternatively spliced variants in various cell lines showed that the long* TLR5 *transcripts are abundantly expressed in nonimmune cells. The ratios of long/short transcripts in human nonimmune cell lines, such as A549, T98G, HaCaT, H460, HEK-293, and Caco-2 cells, and primary mesenchymal stem cells were in the range of 1.25 to 4.31. In contrast, those of human monocytic THP-1 and U937 cells and E6.1 T cells and Ramos B cells were around 0.9. These ratios in human monocytic THP-1 cells were decreased by treatment with IFN-*γ* in a concentration-dependent manner. Based on our findings, we suggest that the newly found long* TLR5* transcripts may be involved in the negative regulation of TLR5 expression and function.

## 1. Introduction

Toll-like receptors (TLRs) are innate immune receptors that consist of an extracellular domain for the recognition of pathogenic components and a cytoplasmic tail with a conserved Toll/IL-1 receptor (TIR) domain for the generation of intracellular signaling [1]. Upon TLR stimulation by pathogenic components, the TIR domain recruits signaling molecules to activate the transcription of diverse genes, including inflammatory and antimicrobial mediators [1]. TLR signaling must be tightly controlled to avoid the overproduction of proinflammatory mediators that would be harmful to the host. As a control mechanism, alternative splicing can be used to modulate the expression and function of TLRs. Members of the TLR signaling pathway are alternatively spliced at a high frequency, producing novel proteins that can change inflammatory outcomes. Alternative splicing has been found in mammalian* TLR* genes and their homologs in plants and* Drosophila* [2–4]. Mouse* TLR4* has two splicing variants that are inducible by interferon-*γ* priming as well as LPS stimulation of primary macrophages [5]. Alternative splicing of human TLR has also been reported. Human* TLR1*,* TLR2*,* TLR3* [6], and* TLR9* [7] have two splicing variants, while* TLR4* has four splicing variants, all of which change the length of the extracellular domain, but their functional significance has not been examined [5]. Alternative splicing of key TLR signaling components, such as* MyD88 *and* IRAK*, has also been reported. A splicing variant of* MyD88*, termed MyD88s, which lacks the intermediate region between the TIR domain and the death domain, inhibits inflammatory signals that are normally mediated by* MyD88* in both mouse [8] and human [9] cells.* IRAK* also has splicing variants. Two splicing variants of murine IRAK2 are inhibitory [10], and a splicing variant of human* IRAK1 *is inhibitory [11]. These studies suggest that the splicing of TLR signaling molecules is involved in the resolution of TLR-directed immune responses.

In the present study, we identified and characterized a new splicing variant in the 5′ untranslated region (5′ UTR) of the human* TLR5* gene.

## 2. Materials and Methods

### 2.1. Cell Culture

Human cell lines, including monocytes (THP-1), T cells (E6.1), keratinocytes (HaCaT), and lung epithelial cells (A549), were purchased from ATCC (Manassas, VA, USA). Human umbilical cord mesenchymal stem cells (MSCs) were obtained from PromoCell (Heidelberg, Germany). The human glioblastoma cell line T98G, monocytes (U937), B cells (Ramos), lung epithelial cells (H460), embryonic kidney epithelial cells (HEK-293), and intestinal epithelial cells (Caco-2) were obtained from the Korean Cell Line Bank (Seoul, Korea). E6.1, Ramos, THP-1, and U937 cells were grown in RPMI-1640 media (Welgene Inc., Daegu, Korea) with 10 mM HEPES buffer (Invitrogen Corp., Gibco BRL, Gaithersburg, MD, USA) and *β*-mercaptoethanol (Invitrogen Corp.). A549 cells and HaCaT cells were grown in DMEM (Welgene Inc.). T98G cells were grown in MEM (Welgene Inc.) supplemented with 1 mM sodium pyruvate solution (Sigma-Aldrich, St. Louis, MO, USA) and MEM Nonessential Amino Acid Solution (Sigma-Aldrich). H460 cells were grown on RPMI-1640 media (Welgene Inc.). HEK-293 cells were grown in DMEM media (Welgene Inc.) with 2 mM L-glutamine (Sigma-Aldrich). Caco-2 cells were grown on DMEM (Welgene Inc.) with 4 mM L-glutamine (Sigma-Aldrich) and 1% nonessential amino acids (Sigma-Aldrich). All media were supplemented with 10% heat-inactivated fetal bovine serum and 1% antibiotic-antimycotic (Invitrogen Corp.). MSCs were grown on MSC growth medium (PromoCell) with 1% antibiotic-antimycotic (Invitrogen Corp.) and 5 *μ*g/ml Plasmocin (InvivoGen, San Diego, CA, USA). The cells were maintained at 37°C in a 5% CO_2_ humidified incubator. To examine TLR5 alternative splicing, THP-1 cells (1 × 10^5^ cells/ml) were treated with IFN-*γ* (Invitrogen Corp.) for 0–24 h.

### 2.2. Primers

**Figure 1:**
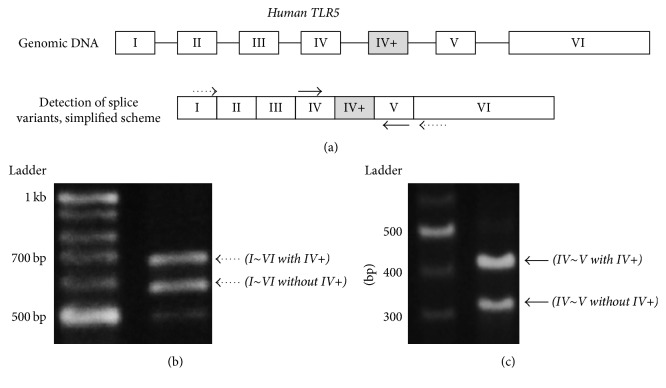
The exon structure of the human TLR5 gene and detection of TLR5 splice variants in human monocytic THP-1 cell line. (a) Human* TLR5* contains seven exons (depicted as boxes). Exons IV+ are alternative (marked by shading). (b) PCR was performed with primers designed to exons I and VI (dotted arrows), or (c) exon IV and exon V (solid arrows). The amplified products were visualized by gel electrophoresis. IV+ designates the newly discovered exon.

To detect* TLR5* alternative splicing, specific primers for RT-PCR were designed to bind to exons I and VI (Figure 1(a), dotted arrows) or exons IV and V (Figure 1(a), solid arrows). To quantify splicing variants of* TLR5*, specific primers were designed for real-time quantitative PCR (RT-qPCR). The primers that recognize the new exon (designated V in Figure 4) were used to detect long* TLR5* transcripts (solid arrows). Short* TLR5* transcripts were detected by primers recognizing the boundary between exons IV and V (dashed arrows). The primers for the* TLR5* reference that recognize exon VII are represented as dotted arrows. The primer sequences are listed in Table 1.

### 2.3. Relative Quantification of Alternative Splicing Variants by Real-Time Quantitative PCR

Total RNA was extracted using the Qiagen RNeasy Kit (Hilden, Germany) according to the manufacturer's instructions. An SD2000 microspectrophotometer (Bioprince, Atlanta, GA, USA) was used to determine RNA concentrations. The cDNA was constructed from 2.5 *μ*g of total RNA using MMLV Reverse Transcriptase (GeneAll, Seoul, Korea) and an Oligo(dT) primer (Invitrogen Corp.) at 50°C for 1 h. The cDNA was amplified by PCR, and the PCR products were stained with Loading Star solution (Dynebio, Seongnam, Korea) and separated on 1.5% agarose gels.

RT-qPCR was conducted using the iQ5 multicolor RT-PCR detection system (Bio-Rad, Hercules, CA, USA) with the iQ SYBR Green Supermix (Bio-Rad). DNA amplification was performed using the primer sequences listed in Table 1, which were designed to specifically identify each of the alternative splicing variants.

Relative quantification of* TLR5* transcripts generated by alternative splicing was performed according to previously established methods [12]. Briefly, in order to determine the relative proportions of* TLR5* transcripts, a “never-spliced” exon of* TLR5* was used as an internal reference, instead of a classical housekeeping gene, with a portion common to the long and short* TLR5* transcripts, that is, exon VII. The real proportions of* TLR5* transcripts were determined according to the principle that the sum of both the long and the short* TLR5* transcripts equals the level of expression of a “never-spliced” TLR5 exon. Therefore, the sum of the ratios of the long TLR5 transcripts to exon VII (denoted [long]) and the short* TLR5* transcripts to exon VII expression ratio (denoted by [short]) must equal 1.0.

### 2.4. DNA Sequencing and Comparative Analysis

*TLR5* PCR products were sequenced by Cosmo Genetech (Seoul, Korea) using the Applied Biosystems 3730 xl DNA Analyzer (Thermo Fisher Scientific Corp., Waltham, MA, USA). BioEdit version 7.2.5, created by Tom Hall (Ibis Biosciences, Carlsbad, CA, USA), was used to assemble the sequencing results. For the identification of amplified DNA fragments, DNA sequences were aligned against the National Center for Biotechnology Information (NCBI) database (NCBI reference sequence: NM_003268.5).

### 2.5. Statistical Analysis

Data were analyzed by one-way analysis of variance (ANOVA) followed by post hoc comparisons with either the Tukey HSD (honestly significant difference test) for groups of data with equal variances or, alternatively, the Games–Howell test for unequal variances using SPSS 12.0 for Windows. Values are expressed as means ± standard deviation (SD). Statistical significance was defined as *p* < 0.05.

## 3. Results

### 3.1. Identification of Human* TLR5* Splicing Variants

**Figure 2:**
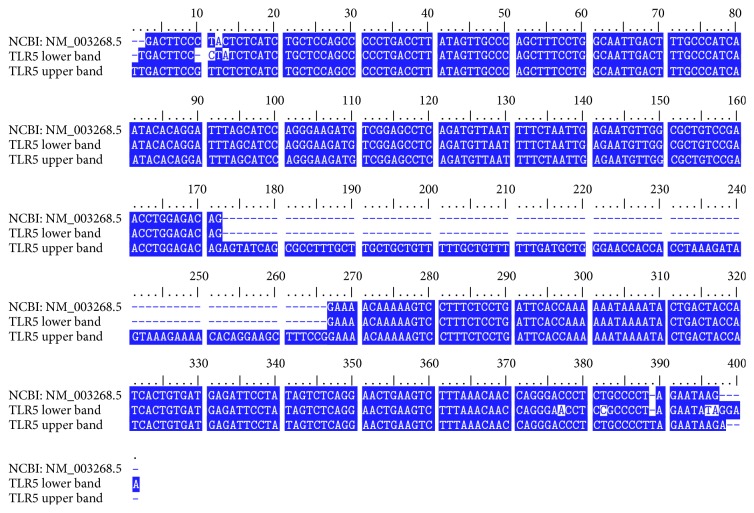
Alignment of sequences of two TLR5 splicing variants with TLR5 sequence from NCBI. Two PCR product bands of the amplification from exon IV to exon VI were collected and sequenced with automated sequencing methods by Applied Biosystems 3730xl DNA Analyzer. These two sequences were then compared to the NCBI* TLR5* sequence (NCBI reference sequence: NM_003268.5) by using BioEdit software version 7.2.5. The detailed sequences are listed in Table 2.

The human* TLR5* gene comprises 6 exons and the coding sequence is in exon VI [13]. In our RT-PCR experiment using primers complementary to exon I and exon VI of* TLR5*, we observed two main bands on the agarose gel. Unexpectedly, a longer product (about 680 bp) was detected in addition to the product of the expected size (about 580 bp), suggesting an alternative splicing variant with an additional exon (Figure 1(b)). To confirm this, a new set of nested PCR primers was designed to amplify the region spanning exons IV through V (Figures 1(a) and 1(c), solid arrows) and two PCR products were detected (Figure 1(c)). A sequence analysis of these products revealed that the longer product contains an additional 94 bp between exon IV and exon V, and its sequences are exactly the same as sequences deposited in the NCBI database (NM_003268.5) (Figure 2). Therefore, our results indicate that human* TLR5* has seven exons and a newly found exon lies between previously reported exons IV and V in the 5′ UTR.

### 3.2. Expression of Human* TLR5* Splicing Variants in Different Cell Lines

**Figure 3:**
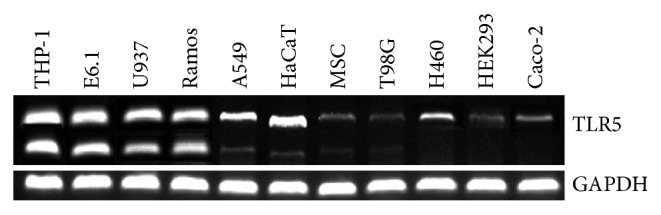
Expression of TLR5 splicing variants in different cell lines. Eleven cell lines were cultured at specific conditions. Cells were collected and 2.5 *μ*g mRNA was used for RT-PCR; 3 *μ*l of each cDNA was used for PCR with alternative splicing detecting primer (35 cycles, 60°C annealing temperature). GAPDH was used as control.

To determine whether two main PCR products can be generated in other human cell lines, we performed an RT-PCR analysis of* TLR5 *expression in various human cell lines using the same PCR primers used in Figure 1(c). As shown in Figure 3, long* TLR5* transcripts were observed in all eleven cell types examined. Short* TLR5* transcripts were clearly detected in immune cells, such as THP-1, E6.1, U937, and Ramos cells, but were weakly detected in nonimmune cells, such as HaCaT, MSC, T98G, H460, HEK-293, and Caco-2 cells.

**Figure 4:**
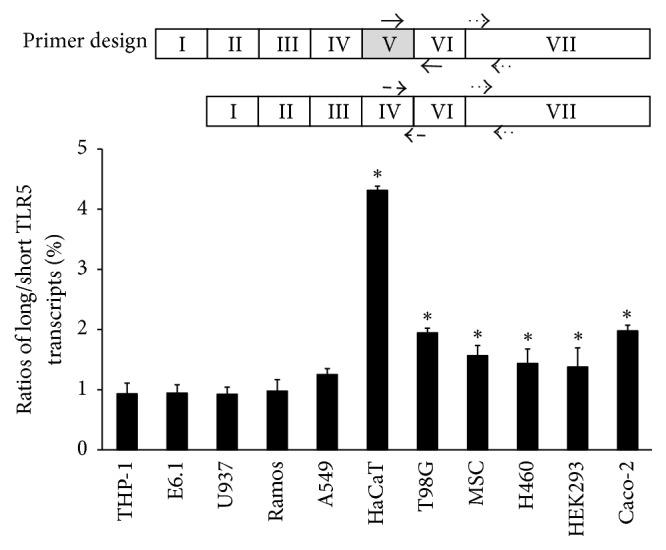
Ratios of long TLR5 transcripts to short TLR5 transcripts in several different cell lines. Expression of long* TLR5* transcripts was quantified by RT-qPCR using primers recognizing exon V (primer pairs were represented by solid arrows), while expression of short* TLR5* transcripts was quantified using primers recognizing the boundary between exons IV and VI (primer pairs were represented by dashed arrows). Exon VII was used as a reference gene (primer pairs were represented by dotted arrows). The expression level of short* TLR5* transcripts was arbitrarily set to 1. *∗* indicates *p* < 0.05 as compared with THP-1.

We determined the precise ratio of long* TLR5* transcripts to short* TLR5* transcripts (long/short* TLR5* transcripts) in each cell line by RT-qPCR, as described in the Materials and Methods. The ratios of long* TLR5* transcripts to short* TLR5* transcripts in human immune cells, such as THP-1, U937, E6.1, and Ramos cells, were around 0.9, and those in nonimmune cells were 1.25 to 4.31 (Figure 4, Table 3).

### 3.3. Regulation of Human* TLR5* Alternative Splicing by IFN-*γ*

**Figure 5:**
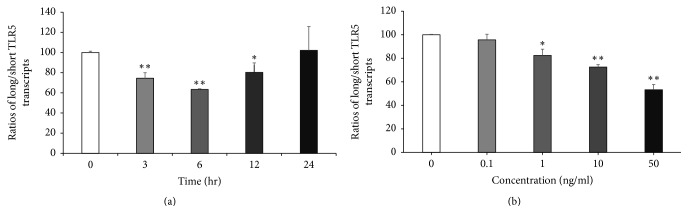
The relative expression of long/short TLR5 transcripts of THP-1 cells upon stimulation with IFN-*γ*. Total RNA was extracted from cells and cDNA was obtained from 2.5 *μ*g total RNA. Quantitative real-time PCR was performed and ΔCt calculation with an internal reference (“never-spliced” exon of* TLR5* gene) was used to normalize data. (a) Cells were stimulated with 10 ng/ml IFN-*γ* for various time points. (b) Cells were stimulated for 6 hours with various concentrations of IFN-*γ*. Bar graphs show relative gene expression ± SD. ^*∗*^*p* < 0.05, ^*∗∗*^*p* < 0.01, *n* = 3.

Since IFN-*γ* is an important activator of macrophages and downregulates TLR5 expression [14], we investigated whether IFN-*γ* influences* TLR5* alternative splicing. THP-1 cells were stimulated with 10 ng/ml IFN-*γ* for 0–24 h and collected for RT-QPCR analysis. The ratio of long/short* TLR5* transcripts began to decline at 3 h, reached 60% of normal ratio at 6 h, and then returned to normal at 24 h after IFN-*γ* treatment (Figure 5(a)). The ratios of long/short* TLR5* transcripts decreased by treatment with IFN-*γ* in a concentration-dependent manner and were reduced by approximately 50% compared to normal levels at a concentration of 50 ng/ml (Figure 5(b)).

## 4. Discussion

In this study, we found a new splicing variant of human* TLR5* with an extra exon; it included 94 nucleotides and was located between previously reported exons IV and V in the 5′ UTR. Since genetic variants of* TLR5* have been clinically associated with disease outcomes such as obesity, type 2 diabetes, and colorectal cancer [15], it is important to determine the functional role of alternative splicing variants of* TLR5*. Based on the sequences in the NCBI database, human* TLRs* have a small number of exons (2–6 exons). The coding regions of* TLR3* and* TLR4* consist of more than two exons, while those of other* TLRs* consist of only the last exon (*TLR1, TLR2, TLR5, TLR6*, and* TLR10*) or the last exon plus a small portion of the second last exon (*TLR7, TLR8*, and* TLR9*). Therefore,* TLR5* is not expected to produce variable proteins, while* TLR3* and* TLR4* are predicted to express a variable protein. In fact, human* TLR4* has four reported splicing variants with different extracellular leucine-rich repeat lengths, but their functional significance has not been evaluated [5]. Although the functional significance of alternative splicing at the 5′ UTR of* TLR* genes remains to be elucidated, it is a common feature of mouse and human* TLR* genes [5], suggesting that alternative splicing of* TLR* genes plays an important role in the precise control of immune activation [16]. Since alternative splicing of the 5′ UTR influences mRNA stability and translation and thus alters the amount of protein translated [17, 18], differential expression levels of alternative splicing variants of* TLR5* in various cell types, especially between immune and nonimmune cells, observed in our study may reflect intrinsic differences of TLR5 protein expression and function between cell types. The preferential expression of long* TLR5* transcripts in nonimmune cells compared to immune cells suggests that the long TLR5 transcripts are selectively induced in nonimmune cells and may function differentially from the short* TLR5* transcripts. IFN-*γ*-induced reduction in the expression of the long* TLR5* transcripts of human monocytes also suggests a different function from that of the short* TLR5* transcripts in activated macrophages.

In summary, we identified a new splicing variant of human* TLR5*. The expression patterns of the splicing variants were different in distinct cell types. Based on our finding that long* TLR5* transcripts were predominantly expressed in nonimmune cells and were significantly reduced by treatment with the proinflammatory cytokine IFN-*γ*, we cautiously speculate that the newly detected long* TLR5 *transcripts may be involved in the negative regulation of the expression and function of TLR5. To confirm this, further studies of the protein expression patterns in different cell types and functional analyses of splicing variants using HEK-293 cells transfected with cloned splicing variants are required.

## Figures and Tables

**Table 1:** Primer sequences.

Name	A/S	Primer sequence (5′ → 3′)	Product size (bp)
Detection primer long TLR5	A	GAGGCGTCCCAGAGTCTC	565
	S	CCTCGTTGTCCTAGCAGAAG	

Detection primer short TLR5	A	GACTTCCCTACTCTCATCTGCT	300
	S	CTTATTCTAGGGGCAGAGGGT	

Quantification primer TLR5 reference	A	CTTCCTGCTCCTTTGATGGC	114
	S	TCCTGATATAGTTGAAGCTCAGC	

Quantification primer long TLR5	A	GAGACAGAGTATCAGCGCCTTTG	134
	S	TTGGTGAATCAGGAGAAAGGACTT	

Quantification primer short TLR5	A	GACTTCCCTACTCTCATCTGCTC	185
	S	GGACTTTTTGTTTTCCTGTCTCCA	

GAPDH	A	ACAGCCTCAAGATCATCAGCAAT	513
	S	AGGAAATGAGCTTGACAAAGTGG	

**Table 2:** * TLR5* splicing variant sequences.

Sequence (5′ → 3′)	
Upper band	TTGACTTCCGTTCTCTCATCTGCTCCAGCCCCCTGACCTTATAGTTGCCCAGCTTTCCTGGCAATTGACTTTGCCCATCAATACACAGGATTTAGCATCCAGGGAAGATGTCGGAGCCTCAGATGTTAATTTTCTAATTGAGAATGTTGGCGCTGTCCGAACCTGGAGACAGAGTATCAGCGCCTTTGCTTGCTGCTGTTTTTGCTGTTTTTTGATGCTGGGAACCACCACCTAAAGATAGTAAAGAAAACACAGGAAGCTTTCCGGAAAACAAAAAGTCCTTTCTCCTGATTCACCAAAAAATAAAATACTGACTACCATCACTGTGATGAGATTCCTATAGTCTCAGGAACTGAAGTCTTTAAACAACCAGGGACCCTCTGCCCCTTAGAATAAGA

Lower band	TGACTTCCCTATCTCATCTGCTCCAGCCCCCTGACCTTATAGTTGCCCAGCTTTCCTGGCAATTGACTTTGCCCATCAATACACAGGATTTAGCATCCAGGGAAGATGTCGGAGCCTCAGATGTTAATTTTCTAATTGAGAATGTTGGCGCTGTCCGAACCTGGAGACAGGAAAACAAAAAGTCCTTTCTCCTGATTCACCAAAAAATAAAATACTGACTACCATCACTGTGATGAGATTCCTATAGTCTCAGGAACTGAAGTCTTTAAACAACCAGGGAACCTCCGCCCCTAGAATATAGGAA

**Table 3:** Relative quantities of TLR5f to TLR5sh isoform in various human cell lines.

	Cell lines										
	THP-1	E6.1	U937	Ramos	A549	HaCaT	T98G	MSC	H460	HEK293	Caco-2
[short]_Q_	0.50	0.48	0.31	0.25	0.46	0.17	0.23	0.39	0.42	0.29	0.41
*α*						**1.2**					
[short]	0.6	0.51	0.32	0.26	0.56	0.20	0.28	0.47	0.50	0.35	0.49

[long]_Q_	0.49	0.48	0.29	0.23	0.64	0.80	0.50	0.67	0.61	0.44	0.89
*β*						**1.07**					
[long]	0.54	0.56	0.34	0.27	0.70	0.87	0.55	0.72	0.67	0.48	0.97

Sum	1.14	1.07	0.66	0.53	1.26	1.07	0.83	1.19	1.17	0.83	1.46

Ratio											
Long/short	**0.94**	**0.95**	**0.92**	**0.98**	**1.25**	**4.31**	**1.94**	**1.57**	**1.43**	**1.38**	**1.98**

[short]_Q_ and [long]_Q_ are the proportions of each transcript over exon VI, calculated by the 2^−ΔCT^ formula. [short] and [long] are the “real” expression proportions. *α* and *β* are the calculated correction factors. The ratios presented here, being expressed over exon IV+, show the ratio of short or long alternative variants. The expression level of short *TLR5* transcripts was arbitrarily set to 1.

## References

[1] Kawai T., Akira S. (2007). TLR signaling. *Seminars in Immunology*.

[2] Haehnel V., Schwarzfischer L., Fenton M. J., Rehli M. (2002). Transcriptional regulation of the human Toll-like receptor 2 gene in monocytes and macrophages. *Journal of Immunology*.

[3] LeBouder E., Rey-Nores J. E., Rushmere N. K. (2003). Soluble forms of Toll-like receptor (TLR)2 capable of modulating TLR2 signaling are present in human plasma and breast milk. *Journal of Immunology*.

[4] Jordan T., Schornack S., Lahaye T. (2002). Alternative splicing of transcripts encoding toll-like plant resistance proteins - What's the functional relevance to innate immunity?. *Trends in Plant Science*.

[5] Wells C. A., Chalk A. M., Forrest A. (2006). Alternate transcription of the Toll-like receptor signaling cascade. *Genome Biology*.

[6] Rock F. L., Hardiman G., Timans J. C., Kastelein R. A., Bazan J. F. (1998). A family of human receptors structurally related to Drosophila Toll. *Proceedings of the National Academy of Sciences of the United States of America*.

[7] Du X., Poltorak A., Wei Y., Bruce B. (2000). Three novel mammalian toll-like receptors: gene structure, expression, and evolution. *Eur. Cytokine Netw*.

[8] Burns K., Janssens S., Brissoni B., Olivos N., Beyaert R., Tschopp J. (2003). Inhibition of interleukin 1 receptor/toll-like receptor signaling through the alternatively spliced, short form of MyD88 is due to its failure to recruit IRAK-4. *Journal of Experimental Medicine*.

[9] Janssens S., Burns K., Vercammen E., Tschopp J., Beyaert R. (2003). MyD88S, a splice variant of MyD88, differentially modulates NF-*κ*B- and AP-1-dependent gene expression. *FEBS Letters*.

[10] Hardy M. P., O'Neill L. A. J. (2004). The murine Irak2 gene encodes four alternatively spliced isoforms, two of which are inhibitory. *Journal of Biological Chemistry*.

[11] Rao N., Nguyen S., Ngo K., Fung-Leung W.-P. (2005). A novel splice variant of interleukin-1 receptor (IL-1R)-Associated kinase 1 plays a negative regulatory role in Toll/IL-1R-induced inflammatory signaling. *Molecular and Cellular Biology*.

[12] Vidal-Petiot E., Cheval L., Faugeroux J. (2012). A new methodology for quantification of alternatively spliced exons reveals a highly tissue-specific expression pattern of WNK1 isoforms. *PLoS ONE*.

[13] Zeng H.-M., Pan K.-F., Zhang Y. (2011). Genetic variants of toll-like receptor 2 and 5, Helicobacter Pylori infection, and risk of gastric cancer and its precursors in a Chinese population. *Cancer Epidemiology Biomarkers and Prevention*.

[14] O'Mahony D. S., Pham U., Iyer R., Hawn T. R., Liles W. C. (2008). Differential constitutive and cytokine-modulated expression of human Toll-like receptors in primary neutrophils, monocytes, and macrophages. *International Journal of Medical Sciences*.

[15] Leifer C. A., McConkey C., Li S., Chassaing B., Gewirtz A. T., Ley R. E. (2014). Linking genetic variation in human Toll-like receptor 5 genes to the gut microbiome's potential to cause inflammation. *Immunology Letters*.

[16] Modrek B., Resch A., Grasso C., Lee C. (2001). Genome-wide detection of alternative splicing in expressed sequences of human genes. *Nucleic Acids Research*.

[17] Rosentiel P., Huse K., Franke A. (2007). Functional characterization of two novel 5′ untranslated exons reveals a complex regulation of NOD2 protein expression. *BMC Genomics*.

[18] Wang G., Guo X., Floros J. (2005). Differences in the translation efficiency and mRNA stability mediated by 5′-UTR splice variants of human SP-A1 and SP-A2 genes. *American Journal of Physiology - Lung Cellular and Molecular Physiology*.

